# Motility of human renal cells is disturbed by infection with pathogenic hantaviruses

**DOI:** 10.1186/s12879-018-3583-x

**Published:** 2018-12-12

**Authors:** Stefan Hägele, Alexander Müller, Christian Nusshag, Jochen Reiser, Martin Zeier, Ellen Krautkrämer

**Affiliations:** 1 Department of Nephrology, University of Heidelberg, Im Neuenheimer Feld 162, 69120 Heidelberg, Germany; 2 Department of Medicine I and Clinical Chemistry/Department of Nephrology, University of Heidelberg, Heidelberg, Germany; 3 Department of Internal Medicine, Rush University Medical Center, Chicago, IL USA

**Keywords:** Hantavirus, Kidney, Tubular epithelium, Glomerulus, Podocytes, Proteinuria

## Abstract

**Background:**

Hemorrhagic fever with renal syndrome (HFRS) caused by pathogenic hantaviruses in Europe and Asia is often characterized by acute kidney injury (AKI) with massive proteinuria. Renal filtration depends on the integrity of epithelial and endothelial monolayers in the tubular and glomerular apparatus. Tubular and glomerular cells represent target cells of hantavirus infection. However, the detailed mechanisms of renal impairment induced by hantaviruses are not well understood.

**Methods:**

We analyzed the cellular consequences of hantavirus infection by measuring adhesion and migration capacity of human renal cells infected with Puumala (PUUV) or Hantaan (HTNV) virus. The impact of hantaviral nucleocapsid proteins (N proteins) on motility was examined by transfection of podocytes.

**Results:**

Infection of kidney cells with hantavirus species PUUV and HTNV causes a significant reduction of migration capacity. The impaired motility depends on viral replication and transfection of podocytes with N protein of PUUV or HTNV reveals that the expression of N protein alone is sufficient to deteriorate podocyte function. The cellular effects are more pronounced for the more pathogenic HTNV than for PUUV that causes a milder form of HFRS.

**Conclusions:**

The direct impairment of migration capacity of renal cells by hantaviral N proteins may contribute substantially to proteinuria observed in the clinical picture of hantavirus infection.

## Background

Hantaviruses are distributed worldwide and among the emerging pathogens that attract the attention of kidney research due to increasing numbers of cases and due to identification of novel species with pathogenic potential to humans [1]. HFRS varies considerably in its symptoms and severity depending on the causative virus species. Severe infections with hantaviruses HTNV, Dobrava-Belgrade virus (DOBV), or PUUV are characterized by AKI with massive proteinuria. The proteinuria is non-selective indicating that glomerular and tubular structures are affected [2]. Moreover, immunofluorescence and electron microscopy studies demonstrate tubular and glomerular changes in renal specimens of patients with hantavirus infection [3–5].

The glomerular filtration barrier mainly exerts its function via three layers: fenestrated endothelium, glomerular basement membrane (GBM), and podocytes. Normal kidney filter function depends on the integrity of GBM and of tubular and glomerular monolayers. Cell adhesion and individual cell motility play a pivotal role in the establishment and maintenance of cell-to-cell contacts, polarity, and renal filtration. Cytoskeletal alterations are characteristic for many renal diseases and changes in motility correlate with proteinuria [6–8]. Glomerular proteinuria is predictive for the severity of hantavirus-induced AKI [9]. The infection of renal cells by hantaviruses may contribute to proteinuria and may represent a determinant in the clinical course of HFRS. Hantaviral N protein was detected in tubular and glomerular cells and cell-to-cell contact structures were changed in biopsy samples of patients with serologically confirmed PUUV infections [3].

However, the underlying mechanisms and effects of hantavirus infection on human renal cells have not been investigated so far. To identify the consequences of hantavirus infection, we analyzed adhesion and motility capacity of human renal cells infected with hantaviruses PUUV or HTNV.

## Methods

### Cells

Primary podocytes (Lonza) were maintained in RPMI medium (Capricorn) supplemented with 10% fetal calf serum (FCS). Human renal epithelial cells (HREpCs) (PromoCell) were cultured in renal epithelial cell growth medium-2 (PromoCell). Human renal glomerular endothelial cells (HRGEnC) (ScienCell) were maintained in endothelial cell medium ECM (ScienCell). Primary cells were only used from passage two to six. The human podocyte cell line was derived from human normal podocytes conditionally transformed with a temperature-sensitive mutant of the simian virus 40 (SV40) large T antigen. Cells were cultured as previously described [10]. Experiments were performed with non-proliferating, differentiated podocytes expressing synaptopodin as podocyte-specific marker of differentiation. Vero E6 cells were maintained in DMEM (Capricorn) supplemented with 10% FCS.

### Viruses and infection

Hantavirus species HTNV strain 76–118 and PUUV strain Vranica were propagated on Vero E6 cells. For infection, cells were incubated with viral inocula at a multiplicity of infection (MOI) of 0.5 and medium was replaced after six hours. MOI was calculated on the respective cell type. On day six after infection, infected cells were quantified by detection of N protein expression via immunofluorescence staining and subjected to the assays.

### Human sera samples

Heat-inactivated (56 °C, 30 min) serum samples of three healthy volunteers and three patients with serologically confirmed PUUV infection were used for incubation of podocytes in migration assays. Semiquantitative proteinuria was determined by urine dipstick: +: 30 mg/dL, ++: 100 mg/dL, +++: 300 mg/dL, and ++++: 1000 mg/dL. Clinical data were collected through a review of medical charts of the Department of Nephrology of the University of Heidelberg, Germany. This study was approved by the Ethics Committee of the University of Heidelberg and it adhered to the Declaration of Helsinki. Written informed consent was obtained from all participants.

### Immunofluorescence (IF)

Cells grown on coverslips were fixed with 3% paraformaldehyde (PFA) and stained with primary and fluorescently-labeled secondary antibodies. The following primary antibodies were used: Mouse anti-N protein PUUV (A1C5, Progen), mouse anti-N protein HTNV (B5D9, Progen). Cell nuclei were stained by Hoechst 33342 (Invitrogen). Images were taken using an Axiocam 506 mono camera attached to an Axio Observer.D1 inverted microscope (Carl Zeiss).

### Western blot

For Western blot analysis, samples were boiled in SDS sample buffer, separated by SDS-PAGE, and transferred to a nitrocellulose membrane. Membranes were blocked with 5% nonfat dried milk in Tris-buffered saline containing 0.1% Tween20 (TBST) for 30 min at room temperature and then incubated for one hour at room temperature with the following antibodies: Mouse anti-N protein PUUV (A1C5, Progen), mouse anti-N protein HTNV (B5D9, Progen). After washing with TBST, the membrane was incubated with near-infrared fluorescently-labeled anti-mouse secondary antibodies (IRDye800, Li-Cor) for one hour at room temperature. After washing with TBST, the membrane was scanned by the Odyssey CLx infrared imaging system (Li-Cor).

### Treatment of viral supernatants

Infectious hantaviral particles in supernatants derived from infected podocytes on day six after infection were inactivated by UV irradiation (1.4 J/cm^2^) with Stratalinker UV Crosslinker equipped with 254 nm UV-light bulbs or depleted by filtration at 5000 g at 4 °C for 2 h through Nanosep centrifugal device with Omega membrane 300 K (Pall Life Sciences) with a molecular weight cut-off of 300 kDa [11].

### Transfection of cells

Podocytes were transfected with pmaxGFP encoding green fluorescent protein (Lonza) together with pCR3.1 (Invitrogen) encoding full-length N protein of PUUV or HTNV using nucleofection (Amaxa Nucleofector 2b device, Lonza) with the Basic Nucleofector Kit for primary mammalian epithelial cells (Lonza). Transfection of pmaxGFP together with empty vector (mock) served as control. After eight hours, cells were subjected to live cell imaging or immunofluorescence. All cells expressing maxGFP co-expressed hantaviral N protein. Percentage and viability of cells expressing max GFP together with N protein did not differ compared to cells transfected with pmaxGFP and empty vector. Amount of N protein expression was quantified by determination of the fluorescence levels of 100 transfected cells that were stained for N protein with anti-N protein antibody. Area, mean fluorescence signal, and adjacent background readings were measured for each cell using ImageJ (v1.52e, NIH). The fluorescence levels were calculated with ImageJ as total corrected cell fluorescence (TCCF) of N protein (TCCF = integrated density – (area of selected cell × mean background fluorescence)) [12].

### Viability assay

Uninfected and infected cells were lysed on day six after infection. The number of viable cells was determined by measuring the amount of ATP using CellTiter-Glo luminescent cell viability assay (Promega).

### Live cell imaging and single cell tracking

Infected and transfected cells (10,000 cells/cm^2^) on μ-slide 2-wells (Ibidi) were subjected to live cell imaging. The motility of infected and uninfected podocytes was monitored for eight hours by JuLi Smart Fluorescence Cell Imager (Digital-Bio). Cells were tracked by the ImageJ manual tracking plugin (Ibidi) and statistical analysis was done by using the chemotaxis tool plugin (Ibidi). The migration of podocytes co-transfected with N protein together with green fluorescent protein (maxGFP; Lonza) was recorded using a Ti-HCS microscope with an Andor Clara interline-CCD-camera (Nikon). Cells expressing maxGFP were tracked and analyzed as described for infected cells.

### Migration assay

Podocytes (45,000 cells/cm^2^) were seeded into μ-plate wells (Ibidi) and infected with hantaviruses. The insert was removed six days after infection and images were taken immediately after insert removal and after eight hours by JuLi Smart Fluorescence Cell Imager (Digital Bio). The average area of migrated cells was measured in three independent experiments. Cells were fixed with 3% PFA and stained for nuclei and hantaviral N protein.

### Adhesion assay

Uninfected or infected podocytes of a single-cell suspension (29,000 cells/cm^2^) were added in each well of a 96-well microtiter plate and left to adhere for one hour at 37 °C. After a triple wash with PBS, adhered cells were fixed, and stained with Sapphire700 (Li-Cor) and DRAQ5 (BioStatus) and quantified via scanning with Odyssey CLx infrared imaging system (Li-Cor).

### Statistical analysis

Data were analyzed using Prism 5.0 (GraphPad Software Inc.). Normal distribution was tested with the Kolmogorov-Smirnov test. Values of two groups were compared using two-tailed Student’s t-test. Values were presented as mean ± standard deviation (SD). *P* values of ≤0.05 were considered significant. *P ≤ 0.05; **P ≤ 0.01; ***P ≤ 0.001; ****P ≤ 0.0001; ns: not significant.

## Results

### Migration capacity of PUUV-infected human primary renal cells

**Fig. 1:**
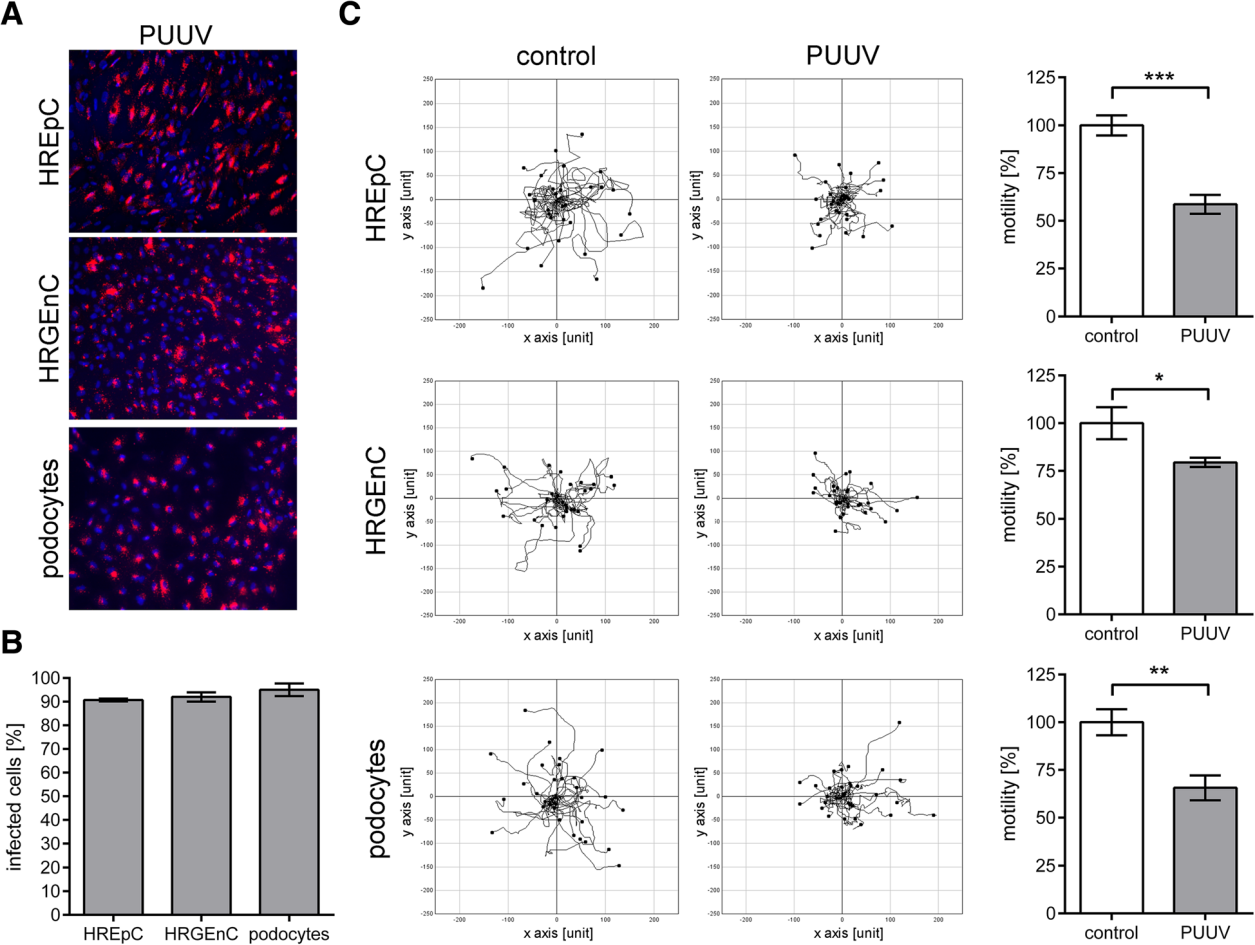
Motility of PUUV-infected human primary HREpCs, HRGEnCs, and podocytes. **a** Infection of renal cells with PUUV. Cells were infected with PUUV at an MOI of 0.5 for six days and stained for hantaviral N protein (red) and nuclei (blue). Cells were imaged at a magnification of × 200. **b** Quantification of infection by detection of N protein expression. **c** Migration of uninfected and infected human primary renal cells was analyzed by single cell tracking via live cell imaging on day six after infection. Three independent experiments were performed. In each single cell tracking experiment 30 cells were analyzed. Shown is mean ± SD

To analyze if hantavirus infection interferes with renal cell function, we measured the motility of PUUV-infected primary HREpCs, HRGEnCs, and human primary podocytes by single cell tracking. Infection was monitored by immunostaining for N protein revealing that more than 90% of cells were positive for PUUV N protein (Fig. 1a and b). Infection of human primary tubular (HREpCs) and glomerular (HRGEnCs and podocytes) cells resulted in an impaired migration capacity as revealed by single cell tracking (Fig. 1c). Motility of infected tubular epithelial cells was reduced to 58.65% ± 2.87% compared to uninfected HREpCs (100% ± 3.05%; *P* = 0.0006). In glomerular cells the levels were reduced to 79.48% ± 1.43% vs. 100% ± 4.86%; *P* = 0.0154 and to 65.66% ± 3.76% vs. 100% ± 3.97%; *P* = 0.0033 for HRGEnCs and podocytes, respectively.

### Migration and adhesion capacity of PUUV- infected podocytes

**Fig. 2:**
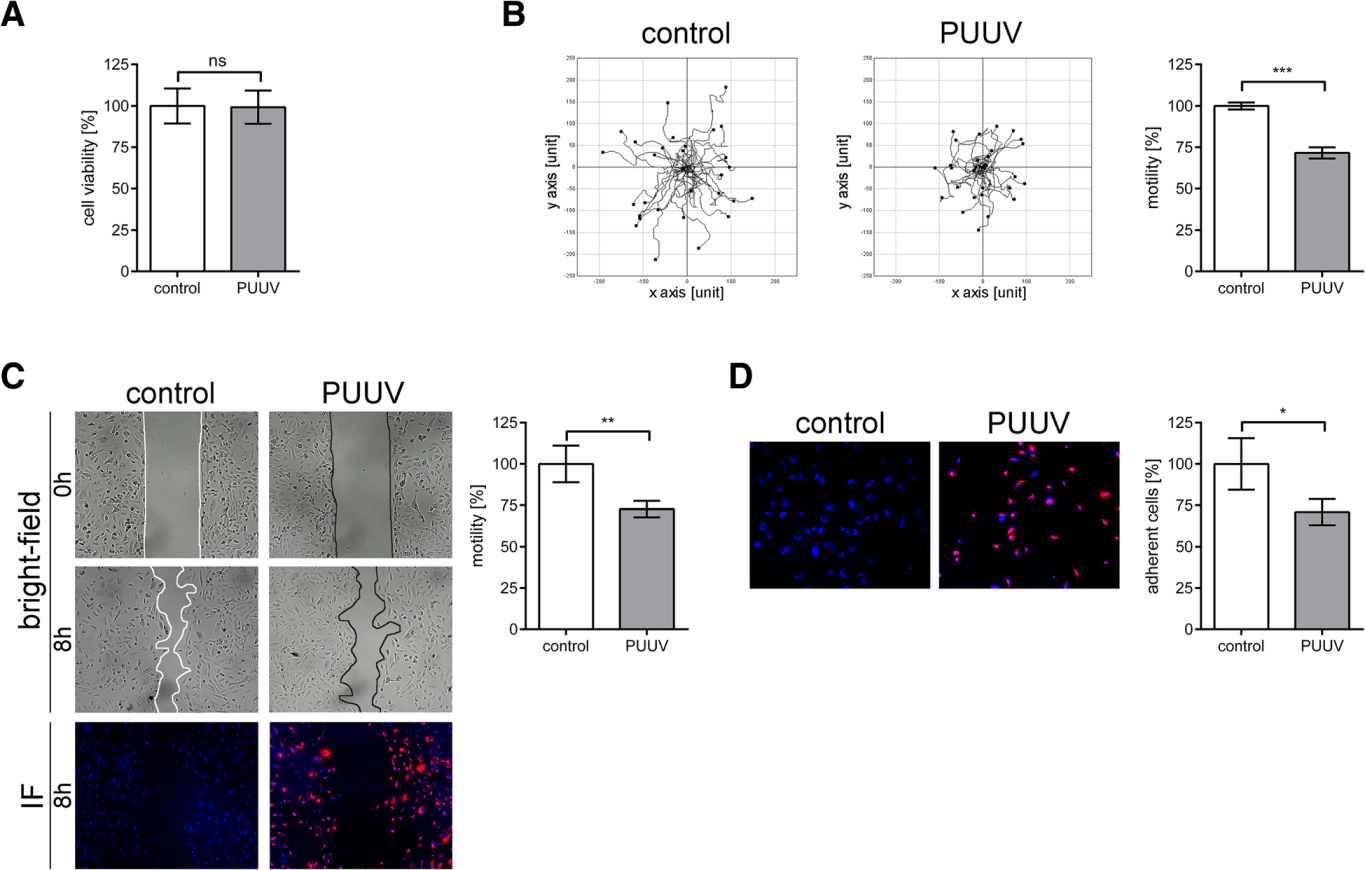
Functional consequences of PUUV-infection in human podocytes. **a** Podocytes were infected with PUUV at an MOI of 0.5 and viability was assessed on day six post infection. Control cells remained uninfected and viability was set to 100%. Three independent experiments were performed in triplicates. Shown is mean ± SD. **b** Migration of uninfected and infected podocytes was analyzed by single cell tracking via live cell imaging. Three independent experiments were performed. In each experiment 30 cells were analyzed. Shown is mean ± SD. **c** For migration assay, podocytes were seeded into μ-plate wells and infected with PUUV at an MOI of 0.5 for six days. After removal of the insert and after eight hours, cell-free areas were measured and relative migration was calculated. Migration of uninfected podocytes was set to 100%. Representative images are shown. Nuclei were stained in blue, N protein in red. Cells were imaged at a magnification of × 100. Three independent experiments were performed. Shown is mean ± SD. **d** Adhesion of PUUV-infected cells. Uninfected and PUUV-infected podocytes were plated into 96 wells and numbers of attached cells were quantified after one hour. Nuclei were stained in blue, N protein in red. Cells were imaged at a magnification of × 200. Adhesion of uninfected cells was set to 100%. Results were obtained from three independent experiments, each performed in quadruplicate. Shown is mean ± SD

Using a human podocyte cell line, we studied the functional consequences of hantavirus infection of renal cells in more detail. We tested the viability of podocytes after infection with PUUV (Fig. 2a). Quantification of infected cells by immunofluorescence of N protein revealed that 93.19% ± 2.29% of cells were infected with PUUV. The infection had no effect on viability. Motility was also analyzed for the PUUV-infected podocyte cell line (Figs 2b and c). The distance covered by infected podocytes was reduced to 73.35% ± 4.24% compared to uninfected cells (100% ± 3.06%; *P* = 0.0003). Measuring cell-free areas by migration assays revealed a migration capacity of infected monolayers that was reduced to 72.80% ± 4.89% vs. 100% ± 11.06%; *P* = 0.0041. In addition, we examined the adhesion of infected cells (Fig. 2d). After infection, the number of adherent cells was decreased to 70.96% ± 7.88% vs. 100% ± 15.53%; *P* = 0.0446.

### Migration and adhesion capacity of HTNV- infected podocytes

**Fig. 3:**
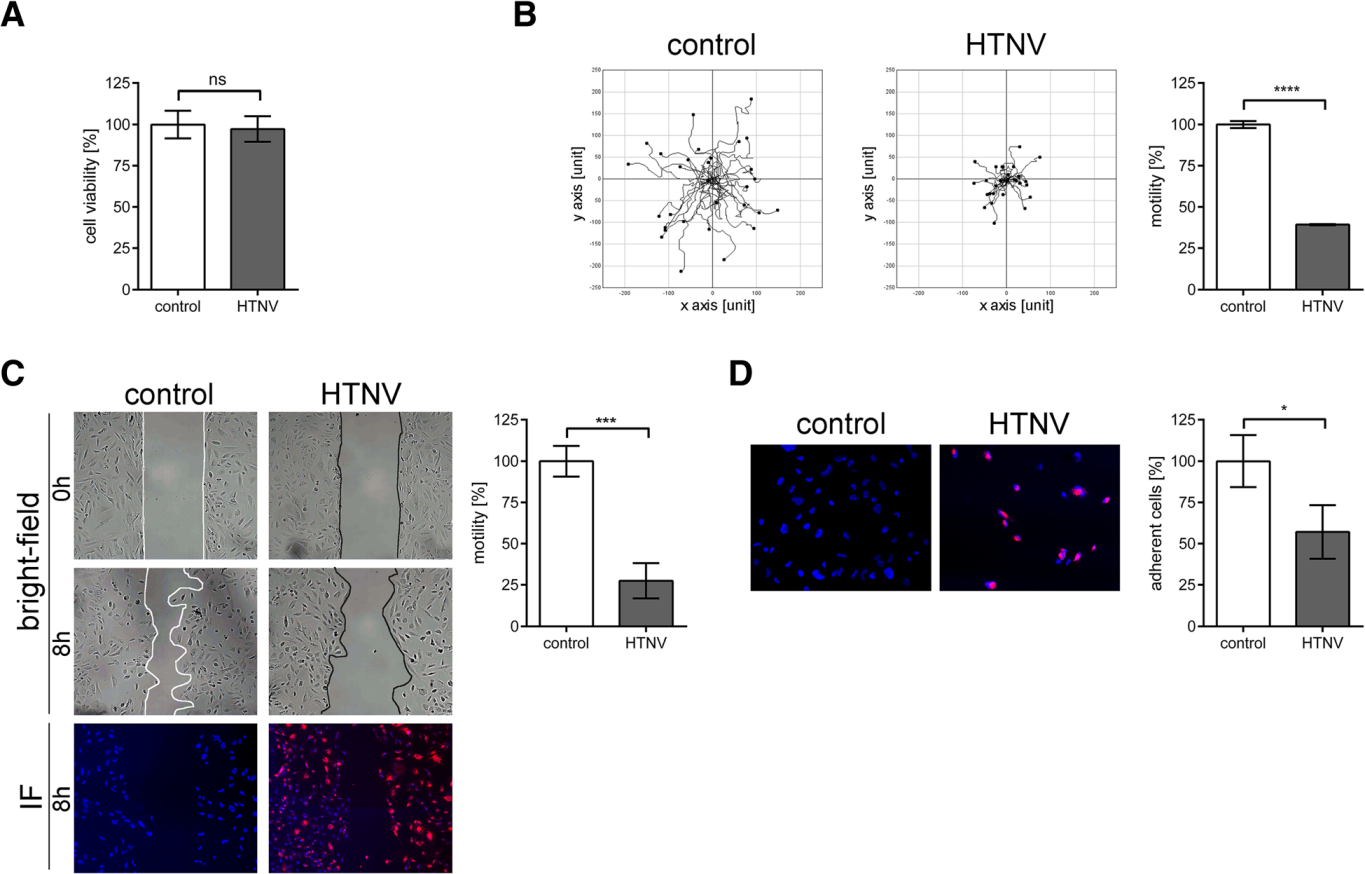
Functional consequences of HTNV-infection in human podocytes. **a** Podocytes were infected with HTNV at an MOI of 0.5 and viability was assessed on day six post infection. Control cells remained uninfected and viability was set to 100%. Three independent experiments were performed in triplicates. Shown is mean ± SD. **b** Motility of uninfected and infected podocytes was analyzed by single cell tracking via live cell imaging. Three independent experiments were performed. In each experiment 30 cells were analyzed. Shown is mean ± SD. **c** Podocytes seeded into μ-plate wells were infected with HTNV and analyzed by migration assay after six days after infection. After removal of the insert and after eight hours, cell-free areas were measured and relative migration was calculated. Migration of uninfected podocytes was set to 100%. Representative images are shown. Cells were imaged at a magnification of × 100. Three independent experiments were performed. Shown is mean ± SD. **d** Uninfected and HTNV-infected cells were plated into 96 wells and numbers of attached cells were quantified after one hour. Results were obtained from three independent experiments, each performed in quadruplicate. N protein was stained in red. Cells were imaged at a magnification of × 200. Adhesion of uninfected cells was set to 100%. Shown is mean ± SD

To investigate whether the effects on podocytes are related to other pathogenic Old World hantaviruses, we examined the infection of podocytes with HTNV (Fig. 3). Quantification of infected cells by immunofluorescence of N protein revealed that 91.76% ± 1.37% of cells were infected with HTNV. Viability of podocytes was not influenced by HTNV infection (Fig. 3a). Motility was impaired compared to uninfected podocytes (Fig. 3b and c). The distance covered by HTNV-infected podocytes was reduced to 40.02% ± 1.92% vs. 100% ± 2.21%; *P* < 0.0001 and migration of infected monolayers was decreased to 27.49% ± 10.69% vs. 100% ± 9.29%; *P* = 0.0009. Adhesion capacity was also reduced in HTNV-infected podocytes (57.13% ± 16.28% vs. 100% ± 15.7%; *P* = 0.0304) (Fig. 3d). The HTNV-mediated deterioration of podocyte functions was more pronounced than the effects caused by PUUV infection.

### Migration of podocytes incubated with culture supernatants and sera

**Fig. 4:**
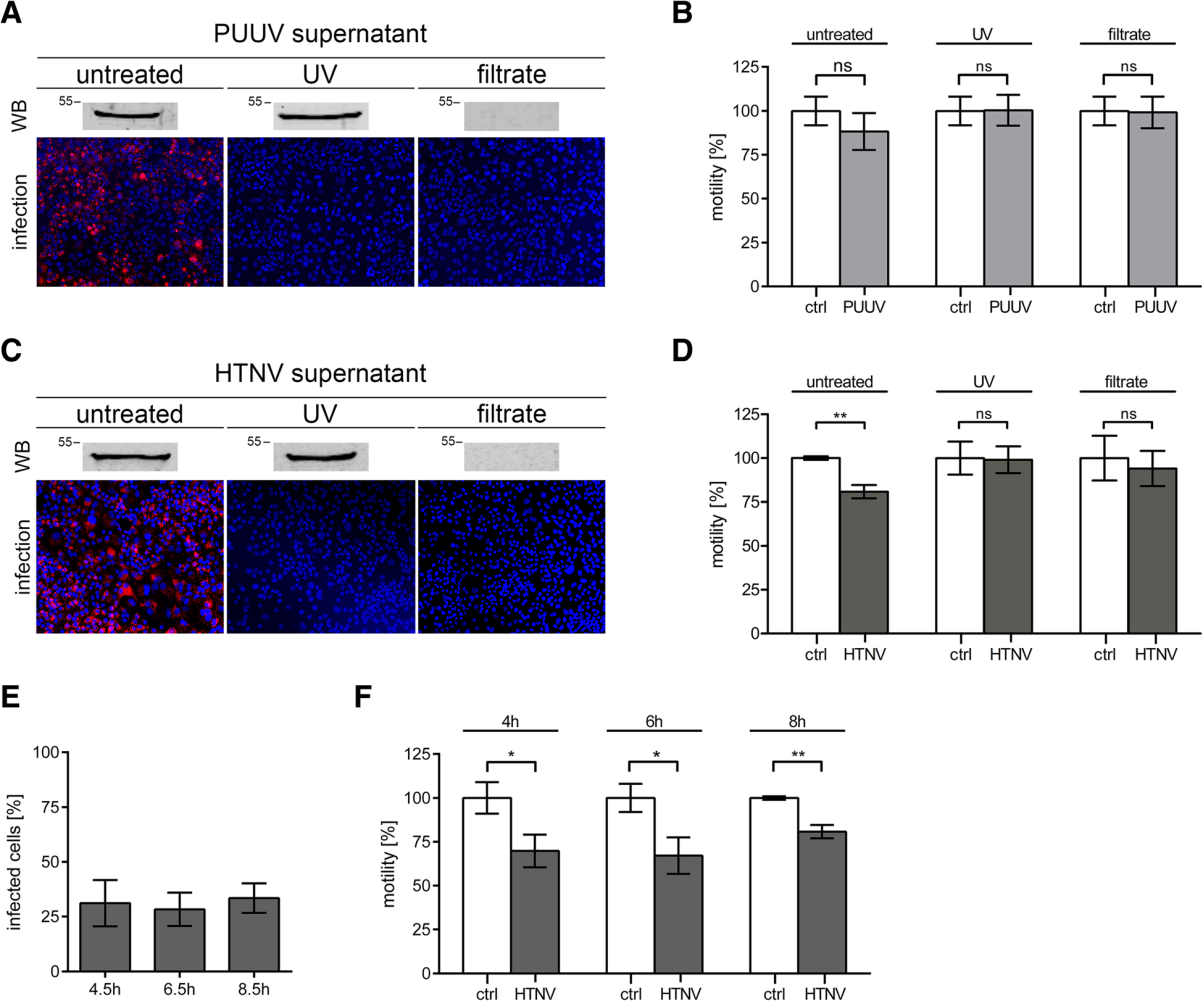
Motility of podocytes incubated with supernatants of infected podocytes. **a** Untreated, UV-irradiated, and filtrated supernatants of podocytes infected with PUUV for six days at an MOI of 0.5 were analyzed for the presence of N protein by Western blot and for infectivity via incubation of Vero E6 cells and subsequent detection of N protein (red). Cells were imaged at a magnification of × 200. **b** Supernatants and filtrates were added to uninfected podocytes 30 min before migration assay. Migration assay was performed in the presence of supernatants and filtrates and analyzed as described in Fig. 2c. Migration of podocytes in the presence of untreated, UV-irradiated, and filtrated supernatants of uninfected podocytes served as control (ctrl) and was set to 100%. (**c** and **d**) Supernatants of HTNV-infected podocytes were treated and analyzed as described for PUUV in (**a** and (**b**). (**e** and **f**) Time course of infection and motility analyzed in podocytes inoculated with untreated supernatants derived from HTNV-infected podocytes after six days of infection. **e** Infection was quantified by counting cells expressing N protein at the indicated time points. **f** Migration assays were started after 30 min of incubation with supernatant and motility was analyzed at the indicated time points. Untreated supernatants of uninfected podocytes served as control (ctrl) and was set to 100%. Results were obtained from three independent experiments. Shown is mean ± SD

Next, we examined whether viral replication is required for the impairment of migration (Fig. 4). To inactivate or deplete infectious PUUV and HTNV particles, supernatants were UV-treated or filtrated. Inactivation and removal of infectious particles were controlled by Western blot analysis of N protein and by inoculation of Vero E6 cells (Fig. 4a and c). N protein was no longer detected in the filtrates and no infection was observed in Vero E6 cells incubated with UV-irradiated or filtrated supernatants. Migration assays with podocytes inoculated with supernatants derived from HTNV-infected podocytes revealed that migration capacity is reduced in the presence of infectious particles. In contrast, incubation with supernatants containing UV-inactivated particles or with filtrated supernatants did not change the migration of uninfected podocytes. No significant effect was observed in samples inoculated with supernatant derived from PUUV-infected podocytes (Fig. 4b and d). Quantification of infection in podocytes incubated with untreated supernatants by detection of N protein after migration assay, revealed that 33.48% ± 6.74% of cells were infected with HTNV and 4.51% ± 3.72% with PUUV after 8.5 h of incubation. The high percentage of HTNV-infected cells may account for the decrease in motility. Therefore, the hantavirus-mediated impairment of podocyte migration depends on viral replication. We examined the levels of infection and decrease of motility at earlier time points after incubation with untreated supernatants collected from HTNV-infected podocytes. 30.20% ± 9.31% of cells were infected at 4.5 h after adding supernatants and the motility was decreased to 69.81% ± 9.32% (Fig. 4e and f). Levels of infected cells and motility impairment did not change over time. The effect is observed very early after infection and does not depend on viral spread.

**Fig. 5:**
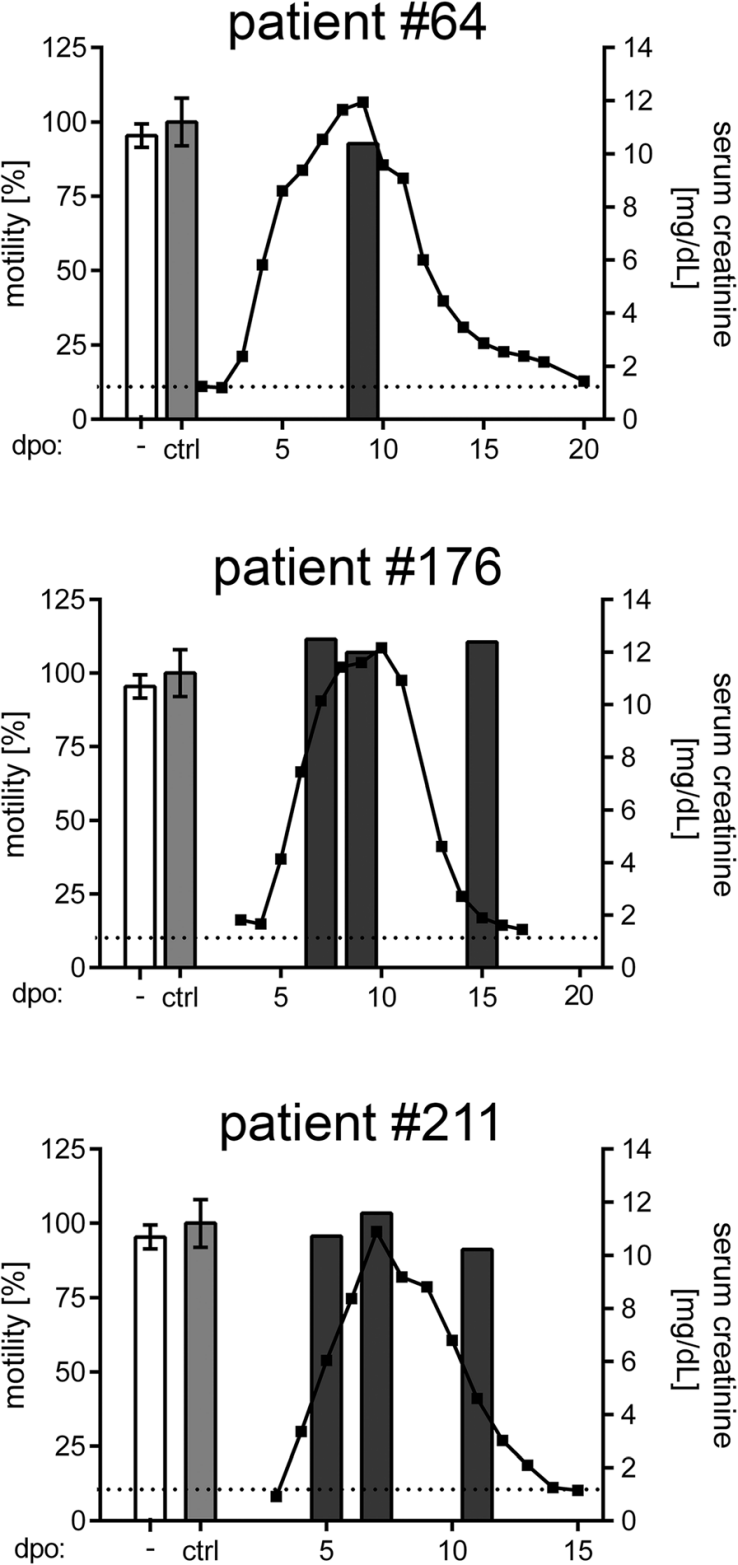
Migration of podocytes inoculated with sera of patients with PUUV infection. Podocytes were inoculated with normal podocyte medium containing 10% FCS (−, white bars), medium containing 50% serum of healthy control individuals (ctrl, light grey bars), or with medium containing 50% serum of patients with PUUV infection collected during the clinical course (dark grey bars) and analyzed by migration assay. Sera were added 30 min before insert removal. Averaged migration of podocytes in the presence of sera derived from three healthy donors was set to 100%. Shown is mean ± SD. Levels of patients’ serum creatinine were shown on the right y-axis to monitor the clinical course of infection. Dashed horizontal line indicates the reference value of serum creatinine for healthy individuals. Dpo: days post onset

We analyzed whether circulating factors affecting podocyte function were present in the serum of patients with acute PUUV hantavirus infection (Table 1, Fig. 5). The patients showed characteristic clinical symptoms (sudden onset of flu-like symptoms), impairment of laboratory parameters (thrombocytopenia, rise in levels of serum creatinine, leukocytes, LDH, CRP, and low levels of serum albumin), proteinuria, and hematuria. Sera samples of the three patients were collected during the clinical course and subjected to migration assays with uninfected podocytes in vitro. Figure 5 shows the course of serum creatinine levels of patients and the migration capacity of podocytes incubated with sera collected at the indicated time points. None of the seven sera sampled at different phases of the clinical course of the three patients demonstrated any impact on the migration of podocytes compared to sera of healthy control individuals or normal cell culture medium. Podocytes incubated with sera from infected patients were not infected as revealed by immunofluorescence analysis of N protein expression (data not shown).

**Table 1:** Characteristics and laboratory parameters of three patients with acute PUUV HFRS^a^

	#64	#176	#211	Reference
Age (years)	37	53	25	
Gender	m	m	m	
Duration of hospitalization (days)	8	15	10	
Max serum creatinine level (mg/dL)	11.94 (9)	12.16 (10)	10.89 (7)	0.1–1.3
Min serum albumin level (g/L)	28 (3)	31 (7)	31.1 (8)	30–50
Max leukocyte count (10^9^/L)	15.6 (3)	15.73 (5)	7.57 (4)	4–10
Min platelet count (10^9^/L)	65 (2)	52 (3)	49 (3)	150–440
Max LDH activity (U/L)	329 (8)	405 (8)	422 (4)	< 248
Max CRP level (mg/L)	94.5 (2)	143.7 (3)	61.2 (4)	< 5
Urine dipstick protein	+++ (2)	+++ (7)	++++ (4)	–
Urinary erythrocyte count (/μL)	25 (3)	69 (3)	43 (5)	0

^a^numbers in brackets indicate the days post onset

### Functional consequences of hantaviral N protein expression

**Fig. 6:**
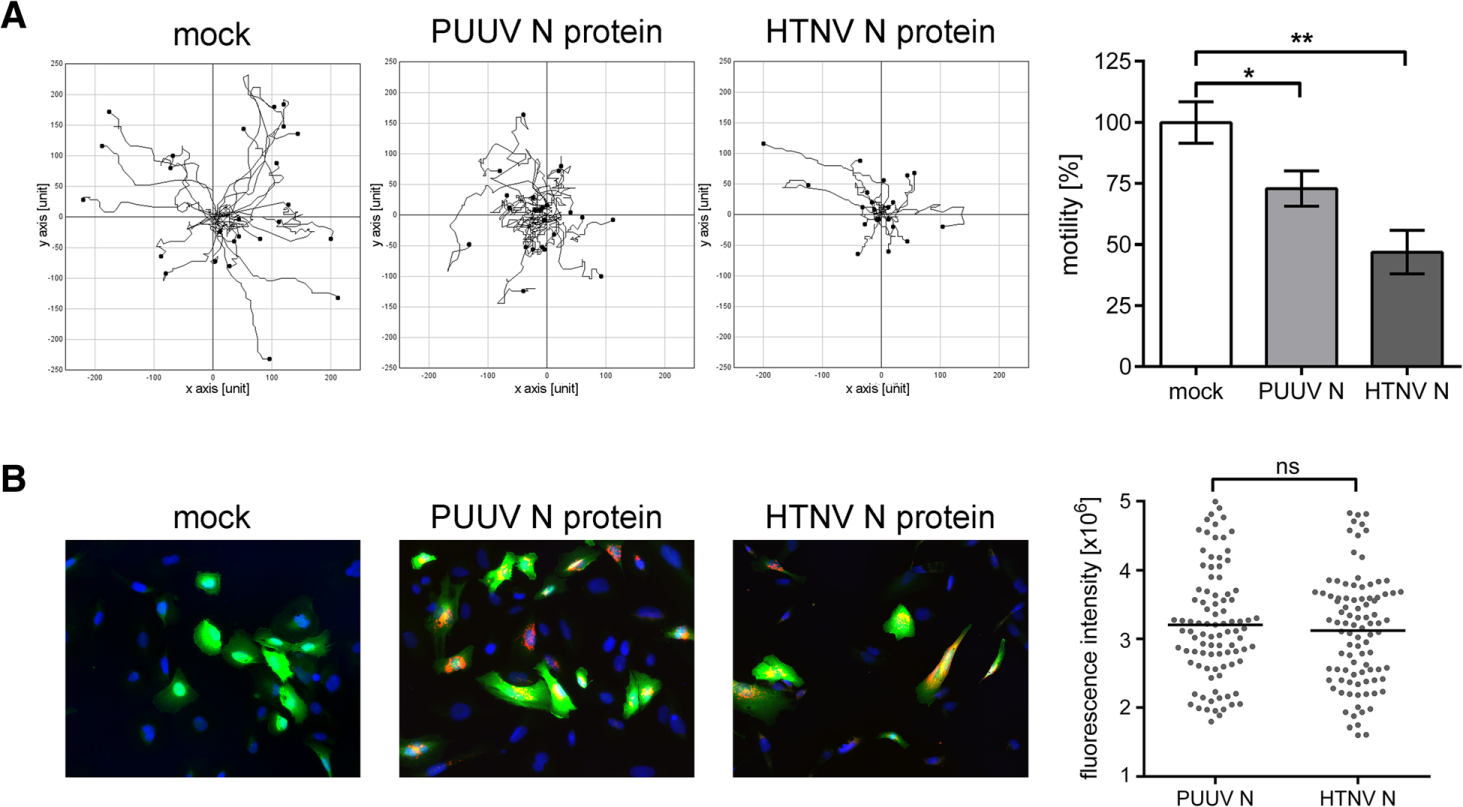
Effect of N protein expression on podocyte migration capacity. **a** Motility of podocytes transfected with plasmids encoding for maxGFP and N protein of PUUV or HTNV was analyzed by live cell imaging eight hours after transfection. Cells transfected with pmaxGFP and empty vector served as control (mock). Three independent experiments with 30 cells quantified in each experiment were performed. Shown is mean ± SD. **b** Transfection efficiency and co-expression of maxGFP and N proteins were monitored by immunostaining for hantaviral N proteins (red). Mean expression level of N proteins was determined by analysis of fluorescence intensity of N protein immunostaining in transfected podocytes. Cells were imaged at a magnification of × 400

The multifunctional hantaviral N protein is the most abundant protein early in infection [13]. Therefore, we wanted to investigate if the expression of N protein is sufficient to induce impairment of podocyte function. We performed single cell tracking of N protein-transfected podocytes by live cell microscopy (Fig. 6). The motility analysis of podocytes expressing recombinant N proteins revealed a reduction of the covered distance to 46.92% ± 8.85%; *P* = 0.0017 for HTNV and to 72.90% ± 7.22%; *P* = 0.0134 for PUUV compared to mock-transfected cells (100% ± 8.45%) (Fig. 6a). Despite equal expression levels of HTNV and PUUV N protein as revealed by determination of fluorescence intensity (Fig. 6b), the effect on podocyte motility was less pronounced for PUUV N protein. Interestingly, the transfection of N protein results in a reduction of motility capacity that is exactly the same level as observed in infection with the respective virus species (Figs. 2b, 3b): the motility of podocytes infected with HTNV was reduced to 40.02% ± 1.92% and motility of podocytes transfected with HTNV N protein was decreased to 46.92% ± 8.85% (*P* = 0.2092). Podocytes infected with PUUV exhibited a reduced motility of 73.35% ± 4.24% and motility of podocytes expressing PUUV N protein was reduced to 72.90% ± 7.22% (*P* = 0.7876).

Together, these results demonstrate that the expression of hantaviral N proteins alone is sufficient to induce impairment of podocyte motility in a virus-specific manner.

## Discussion

Damage to host organisms by pathogens may indirectly occur via the immune system and/or directly due to interference with cellular processes during their replication cycle. The direct effects are often cell type-specific and mediated by modulation of structural components or interference with signaling pathways of the host cell. Hantaviruses infect different organs and cell types. Cells of the respiratory tract, of the kidney, and of the immune system have been described to be susceptible to hantavirus infection [3, 14–17]. In most cases of infections with Old World hantaviruses, renal involvement dominates and leads to AKI, proteinuria, and microhematuria. The underlying molecular and cellular mechanisms in the pathogenesis of AKI and leakage are not completely understood. Direct effects observed in infected renal cells such as disruption of cell-to-cell contacts and alterations in motility and adhesion contribute to proteinuria. However, the role of direct infection of target cells in the failure of organs in HCPS and HFRS needs to be further investigated.

We demonstrate that hantaviruses disturb cellular function of human primary renal cells that may result in proteinuria. Infection of both, tubular and glomerular cell types, leads to severe alterations in motility. The expression of hantaviral N protein alone is sufficient to impair migration. N protein may represent a pathogenicity factor of hantavirus-induced proteinuria. An association of structural changes and hantaviral N protein expression has also been described in tubules of patients with acute hantavirus infection [3].

Neither non-infectious supernatants derived from infected podocytes nor sera from patients with hantavirus infection exhibit any effect on podocytes in vitro. Therefore, viral replication is required to impair cellular motility. In addition, local effects of soluble factors released by renal or immune cells may contribute to the clinical course in vivo. It is also possible that infection sensitizes renal cells to soluble factors, as it is described for hantavirus-infected HUVECs and VEGF [18]. Cytokines may act on these cells and contribute or counteract to motility alterations. Several signal cascades have been described to be involved in the regulation of podocyte function: vascular endothelial growth factor (VEGF), urokinase plasminogen activator receptor (uPAR), or interleukin-6 (IL-6) are modulators of podocyte signaling [7, 19, 20]. Interestingly, these mediators are also known to be up-regulated in hantavirus infection [21–24]. In cells sensitized by infection, these effectors may contribute or counteract to the impairment of motility mediated by N protein expression. Further studies are necessary to resolve the role of direct and indirect effects of hantavirus infection in the balance of podocyte motility and transient proteinuria.

The functional consequences of infection and N protein expression were more pronounced for HTNV than for PUUV. These observations from in vitro experiments correspond to the clinical picture of PUUV- and HTNV-induced HFRS with HTNV being the more pathogenic virus species [25]. However, it remains to examine if the observed cellular effects of renal infection contribute to the severity of the clinical course of PUUV and HTNV infection. The underlying mechanisms for differences in hantaviral pathogenicity are not fully understood. Analysis of reassortants between the weakly pathogenic DOBV-Aa subtype and the highly virulent subtype DOBV-Af revealed that characteristics concerning induction of the innate immune system in host cells are determined by the segments encoding for N protein and RNA-dependent RNA polymerase [26]. Studies with New World hantavirus Andes virus (ANDV) indicated that the N protein of ANDV interferes with signaling cascades of the innate immune system [27, 28]. Gorbunova et al. demonstrated that the ANDV N protein enhances permeability of microvascular endothelial cells via activation of RhoA [29]. These findings and our results show that N proteins of New and Old World hantaviruses interfere with cellular signaling and function and may act as a pathogenicity factor in hantavirus infection.

Hantaviral N proteins share high homology between species and it would be of great interest to identify N protein-specific characteristics responsible for the differences in the functional outcome. Future work will focus on the mode of interaction of different hantaviral N proteins with specific target cells. The analysis of the underlying mechanisms of N protein-induced cellular alterations will help to understand the pathogenesis of renal impairment caused by hantavirus infection.

## Conclusions

Acute kidney injury with massive proteinuria is a hallmark of HFRS. The mechanisms of renal impairment are not completely understood. Hantaviruses PUUV and HTNV infect cells of the glomerular and tubular apparatus. We showed that infection of renal cells results in severe changes in the motility capacity. Furthermore, our results demonstrate an important role of hantaviral N protein in these effects. The identification of N protein as a pathogenicity factor provides useful insights in the possible mechanism of proteinuria in HFRS.

## Abbreviations

AKI: Acute kidney injury
ANDV: Andes virus
CRP: C-reactive protein
DOBV: Dobrava-Belgrade virus
dpo: Days post onset
GFP: Green fluorescent protein
HFRS: Hemorrhagic fever with renal syndrome
HREpC: Human renal epithelial cell
HRGEnC: Human renal glomerular endothelial cell
HTNV: Hantaan virus
IF: Immunofluorescence
LDH: Lactate dehydrogenase
MOI: Multiplicity of infection
PFA: Paraformaldehyde
PUUV: Puumala virus
SD: Standard deviation
